# Higher Versus Lower Mean Arterial Blood Pressure Targets in Vasodilatory Shock

**DOI:** 10.1111/acem.70240

**Published:** 2026-02-12

**Authors:** Brit Long, Michael Gottlieb

**Affiliations:** ^1^ Department of Emergency Medicine University of Virginia School of Medicine Charlottesville Virginia USA; ^2^ Department of Emergency Medicine Rush University Medical Center Chicago Illinois USA

**Keywords:** critical care, mean arterial pressure, mortality, norepinephrine, renal replacement therapy, sepsis, shock, vasodilatory shock, vasopressors

**Table:** 

NNT color recommendation	Black (Harms > Benefits)
Summary heading	Higher MAP targets are likely associated with increased 28‐day all‐cause mortality, less RRT‐free days, and increased risk of arrhythmia
Benefits in NNT	No one was helped (28‐day all‐cause mortality, 90‐day all‐cause mortality, RRT requirement, RRT‐free days, and risk of arrhythmia)
Benefits in percentages (absolute risk reduction)	No one was helped
Harms in NNT (NNH)	1 in 29 were harmed (increased 28‐day all‐cause mortality)
	1 in 68 were harmed (increased risk of arrhythmia)
	No one was harmed (RRT requirement)
	1.64 days decrease in RRT‐free days
	No one was harmed (90‐day all‐cause mortality)
Harms in percentages	3.4% higher risk of 28‐day all‐cause mortality with higher MAP targets
	1.5% higher risk of arrhythmia with higher MAP targets
	No change in RRT requirement
	1.64 days decrease in RRT‐free days
	No change in 90‐day all‐cause mortality
Efficacy endpoints	28‐day all‐cause mortality, 90‐day all‐cause mortality, RRT‐free days, RRT requirements
Harm endpoints	28‐day all‐cause mortality, 90‐day all‐cause mortality, RRT‐free days, RRT‐requirement, and adverse reactions
Who was in the studies	Four randomized trials with 3873 participants ≥ 18 years of age with vasodilatory shock requiring vasopressor support

Abbreviations: MAP—mean arterial pressure; RRT—renal replacement therapy.

## 1 Narrative

Vasodilatory shock is associated with a variety of etiologies and can lead to significant morbidity and mortality. Ensuring appropriate end‐organ perfusion with an adequate mean arterial pressure (MAP) is an important component of resuscitation. This may necessitate the use of vasopressors, but while vasopressors can improve perfusion and MAP, they may be associated with toxicity and adverse events [1]. The 2021 Surviving Sepsis Campaign guidelines recommend targeting a MAP of 65 mmHg [2]. Higher MAP targets may improve outcomes in those with vasodilatory shock, particularly in elderly patients and those with chronic hypertension. However, prior randomized controlled trials (RCTs) and meta‐analyses have not conclusively demonstrated the benefits or harms of this approach, and a specific MAP target remains controversial [3, 4, 5, 6, 7].

The meta‐analysis summarized here included RCTs of participants ≥ 18 years of age with vasodilatory shock requiring vasopressor support [8]. The primary outcomes included 28‐day and 90‐day all‐cause mortality rate. Secondary outcomes included renal replacement therapy (RRT) requirement, RRT‐free days, and safety outcomes (defined as arrhythmias, myocardial injury, bleeding, gastrointestinal ischemia, limb ischemia). The authors performed prespecified subgroup analyses for the primary outcome based on age (< 65 years versus ≥ 65 years), chronic hypertension, and septic shock etiology. The systematic review prespecified a primary frequentist analysis in the registered protocol and complemented it with Bayesian hierarchical meta‐analysis. Of note, a Bayesian hierarchical meta‐analysis combines results from multiple studies while accounting for differences between them. Each study contributes its own estimate, and the model assumes these study effects come from a shared underlying distribution. This approach naturally handles between‐study variation and uncertainty. The model generates a pooled estimate with clear probability‐based uncertainty. In this analysis, probabilities are reported with 95% credible intervals (CrIs) instead of 95% confidence intervals (CIs) reported in frequentist statistics. In frequentist statistics, the 95% CI means that if this experiment were repeated many times, 95% of the calculated intervals would contain the true, fixed parameter. In Bayesian statistics, the 95% CrI means that there is a 95% probability that the true parameter value lies within this specific interval.

The systematic review and meta‐analysis included four trials (*n* = 3873 participants) published between 2014 and 2025. Trials were conducted in the intensive care unit setting in Canada, Japan, France, the United Kingdom, and the United States [8]. Definition of higher and lower MAP targets varied in the included trials, ranging between usual care (defined as median of 72–73 mmHg in the “65 Trial”) up to 85 mmHg for the higher target and 60–70 mmHg for the lower target [4]. Septic shock was the most common cause of vasodilatory shock (48%–100%). Norepinephrine was the most commonly utilized vasopressor and was used for longer periods of time in the higher MAP groups. The OPTPRESS trial was the only trial with protocolized vasopressin use [9]. The achieved MAP was lower in the lower MAP group compared to the achieved MAP in the higher MAP group, but the values in the lower MAP group still surpassed the predefined targets. The actual MAP separation between the groups ranged between 5 and 15 mmHg. There was no difference in fluid administration, steroid use, or urine output between the groups.

Higher MAP targets increased the risk of 28‐day all‐cause mortality (risk ratio [RR]: 1.10, 95% CI: 1.01–1.19, absolute risk increase: 3.4%, number needed to harm [NNH]: 29). There was no statistically significant increase in 90‐day all‐cause mortality.

Bayesian hierarchical meta‐analysis estimated a median RR of 1.18 (95% CrI: 1.003–1.257) for higher versus lower MAP targets concerning 28‐day mortality, with a posterior probability of harm (RR > 1) of 97.8%. For 90‐day all‐cause mortality, the Bayesian hierarchical model estimated a median RR of 1.15 (95% CrI: 1.013–1.240), with a posterior probability of 98.7%.

Higher MAP targets reduced the number of RRT‐free days (mean difference: −1.64 days, 95% CI: −3.05 to −0.23 days) and increased the risk of cardiac arrhythmias (RR: 1.32, 95% CI: 1.01–1.72, absolute risk increase: 1.5%, NNH: 68). This risk of cardiac arrhythmia was primarily driven by supraventricular arrhythmias (RR: 1.71, 95% CI: 1.12–2.61), with no difference in ventricular arrhythmias (RR: 1.02, 95% CI: 0.62–1.68). There was no difference in RRT requirement. There was also no significant difference in myocardial injury, bleeding, gastrointestinal ischemia, or limb ischemia.

Subgroup analysis revealed increased mortality with higher MAP targets in patients ≥ 65 years (RR: 1.17, 95% CI: 1.02–1.34), but this effect did not exist (was not statistically significant) in patients < 65 years, septic shock, chronic hypertension, or non‐hypertensive patients. In patients with chronic hypertension, higher MAP targets were associated with reduced RRT use (RR: 0.83, 95% CI: 0.71–0.98).

## 2 Caveats

While this meta‐analysis has several strengths, including a prospectively registered protocol, there are important considerations [8]. First, there were only four RCTs included, with one study contributing nearly two‐thirds of participants which could significantly influence the pooled effect estimates [4]. Second, studies used an open‐label design, which can introduce performance bias, though studies evaluated all‐cause mortality as the primary outcome, which can mitigate this bias. While most trials primarily enrolled patients with septic shock, the 65 Trial and OVATION included broader vasodilatory shock [4, 10]. There was also significant heterogeneity regarding the definition of “higher” versus “lower” in the included studies, with the 65 Trial utilizing usual‐care for the higher MAP target arm versus a target range such as 80–85 mmHg as used in OPTRESS and SEPSISPAM [3, 4, 9]. However, the lower MAP targets varied, including 60–65 mmHg and 65–70 mmHg, and this latter range may not truly be in fact “lower.” There was significant variation in the duration of vasopressors, and the OPTPRESS trial included the protocolized early use of vasopressin [9]. The time of endpoint ascertainment varied across studies, which may also contribute to heterogeneity. Another limitation is the inability of the meta‐analysis to evaluate the heterogeneity of treatment effects, as the available trial‐level data do not allow for detailed subgroup analyses based on individual patient characteristics. Individual patient phenotypes may have varying clinical and physiological responses to differing MAP targets. However, overall heterogeneity for the primary outcome was very low (*I* ^*2*^ = 0%).

While the meta‐analysis graded the certainty of evidence for the primary outcome as “moderate”, this is likely overly generous. Per the GRADE criteria, high certainty indicates that there is a high level of confidence in the effect estimate and that future studies are unlikely to change this estimate [11]. Moderate certainty implies there is sufficient evidence to support a conclusion, but future studies may impact this confidence. Low certainty means that there is limited evidence and that the true effect could be substantially different from the estimate. Very low certainty indicates that there is insufficient evidence to support firm conclusions. Both the SEPSISPAM and OPTRESS trials were rated as having several concerns for bias, with SEPSISPAM having deviations from the intended interventions with frequent clinician overrides of the high‐target strategy and OPTRESS having concerns for potential overestimation of the effect size after early termination for ineffectiveness [3, 9]. The OVATION trial had several concerns for bias due to issues with adherence to the intervention, with high crossover rate (27%) and prolonged time outside the assigned MAP targets (over 38% of the time) [10]. Thus, a rating of low certainty of evidence is likely more reflective of the meta‐analysis data. Finally, this meta‐analysis and the included trials did not utilize individualized hemodynamic management and tissue‐perfusion endpoints (e.g., lactate clearance, urine output) [8]. Individualized hemodynamic management tailors cardiovascular support to a specific patient rather than using fixed targets. Clinicians adjust fluids, vasopressors, inotropes, and ventilator settings based on the patient's physiology, comorbidities, and response to therapy.

In summary, the meta‐analysis discussed here found that higher MAP targets in adults with vasodilatory shock increased 28‐day mortality, had fewer RRT‐free days, and a higher risk of arrhythmia. Thus, we have assigned a color recommendation of Black (Harm > benefit) for higher MAP targets in those with vasodilatory shock. Further studies are required to evaluate other vasodilatory phenotypes and the use of individualized hemodynamic management and tissue‐perfusion endpoints [12, 13, 14].

## Disclosure

No AI program was utilized in the construction of this manuscript.

## Conflicts of Interest

The authors declare no conflicts of interest.

## Data Availability

Data sharing is not applicable to this article, as no datasets were generated or analyzed during the current study.
